# Retraction: IL-1β/HMGB1 signalling promotes the inflammatory cytokines release via TLR signalling in human intervertebral disc cells

**DOI:** 10.1042/BSR20160118_RET

**Published:** 2026-07-24

**Authors:** Fang Fang, Dianming Jiang

**Affiliations:** Department of Orthopaedics, the First Affiliated Hospital of Chongqing Medical University, 400016, Chongqing, China

**Keywords:** high mobility group box 1 (HMGB1), interleukin (IL)β, intervertebral disc degeneration (IDD)

This article is being retracted from *Bioscience Reports* at the request of the Editor-in-Chief and the Editorial Board. This follows an internal investigation, which alerted the Editorial Board to numerous similarities that exist between figures of this article and those published by different author groups. Specifically, these similarities include the following:

- Duplications identified in Figure 3C
  ○ Between the first two GAPDH bands of this article and the last two B-actin bands from Figure 4B of Qin et al. 2016 (DOI: 10.1042/BSR20150271)
  ○ Between the second and third MMP-3 bands of this figure panel
  ○ Between the fourth MMP-9 band of this article and the first KLF-4 band from Figure 1C Sun et al. 2015 (DOI: 10.1155/2015/473742).
  ○ Some evidence of manipulation has been noted within the MMP-1, MMP-9 and TIMP-1 bands.
- Duplications identified in Figure 5B
  ○ Between the GAPDH bands of this article and the Figure 6B GAPDH bands from Li et al. 2017 (DOI: 10.1042/BSR20160445)
  ○ Between the first two TLR-4 bands of this article and the Figure 1C second and third CXCR4 bands from Yu et al. 2017 (DOI: 10.1042/BSR20170122)

The authors have been contacted with regard to the retraction and have not responded to the Journal's queries or the concerns raised. Given the extent of the issues raised, the Editorial Board stands by the decision to retract the article.

